# Advancing the Achieve Catheter in Different Branches of Pulmonary Vein as a Routine Approach During Cryoballoon Ablation of Atrial Fibrillation

**DOI:** 10.1155/crp/7868137

**Published:** 2026-07-29

**Authors:** Mingyu Sun, Zhen Jin, Ming Liang, Zulu Wang, Zhiqing Jin

**Affiliations:** ^1^ National Key Laboratory of Frigid Zone Cardiovascular Diseases (NKLFZCD), Department of Cardiology, General Hospital of Northern Theater Command, Shenyang, China, syjqzyy.com

**Keywords:** atrial fibrillation, catheter ablation, cryoablation, cryoballoon, pulmonary vein isolation

## Abstract

**Background:**

Cryoballoon ablation (CBA) for pulmonary vein isolation (PVI) has demonstrated high procedural success rate and promising clinical outcome. How to expand the antrum ablation area and get better results is a challenging topic for patients with paroxysmal and persistent AF.

**Methods and Results:**

We enrolled 709 patients with nonvalvular AF who underwent initial PVI with a second‐generation CB between January 2020 and August 2022, including 293 CBA applications in different branches of each PV and 416 in the same branch of each PV. The orientation difference in the transverse plane was negatively correlated with the ostium–bifurcation distance. In the different‐branch CBA group, after the first application in one branch, PV potentials could still be recorded in the other branch in 39 of the 293 patients (13.3%) and the PV foci could trigger AF in 7 of them. In a consecutive 30 patients by using 3D mapping system, the total antral surface lesions after the application in a second branch were larger than those after the first application (*p* < 0.001). The fluoroscopy time increased in the different‐branch CBA group. The recurrence rate of atrial arrhythmia was statistically lower in the different‐branch CBA group compared with that in the same‐branch CBA group (*p* = 0.034) at a 2‐year follow‐up.

**Conclusion:**

CBA applications performed in different branches of each PV could create wide isolated antral surface areas and eliminate PV foci adequately. CBA in different branches of PVs with long ostium–bifurcation distances had less significance. The recurrence rate of atrial arrhythmia was statistically lower in the different‐branch CBA group compared with that in the same‐branch CBA group.

## 1. Introduction

Cryoballoon ablation (CBA) has become an established therapeutic option to obtain pulmonary vein isolation (PVI), which is the cornerstone of atrial fibrillation (AF) ablation [1, 2]. CBA has demonstrated high success rates of procedures and promising clinical outcomes in patients with paroxysmal and persistent AF [3, 4]. In radiofrequency catheter ablation (RFCA), the ablation line could be designed according to the pulmonary vein (PV) anatomy, and a larger isolation area is associated with a lower recurrence rate and a better clinical outcome [5, 6]. The PV anatomy differs in various patients, and the lesion of the CBA might be influenced by the PV anatomy and the limited balloon size and morphology. CBA in the same branch of each PV for one or two times is the most conventional approach. In some cases, advancement of the CB in different branches of PV was used during the segmental ablation procedure [7]. In the present study, we aimed to explore whether advancing the CB in different branches of PVs as a routine approach would expand the antrum ablation area and lead to better outcomes.

## 2. Methods

### 2.1. Study Population

In the present study, we enrolled 293 consecutive patients with highly symptomatic, drug‐refractory paroxysmal or persistent AF who underwent an initial PVI in different branches of PVs with a second‐generation CB (Arctic Front Advance, Medtronic, Inc., Minneapolis, MN, USA) as a routine approach between January 2020 and August 2022. The 416 contemporary cases undergoing conventional CBA in the same branch of PVs in the other ward were included as the control group. Exclusion criteria were left atrium (LA) diameter > 55 mm, acute coronary syndrome, intracardiac thrombi documented by transesophageal echocardiography, and stroke or transient ischemic attacks in the previous 1 month, requirement of RFCA of concomitant arrhythmias. The study protocol was approved by the hospital’s institutional review board and written informed consent was obtained from all patients.

### 2.2. The Method for Measuring the PV Protocol

The CT examinations were reviewed retrospectively at a GE Advanced workstation 4.5. The ostial diameters of branches of PV and ostium–bifurcation distance measurements were generated independently by two experienced radiologists. A cross section perpendicular to the long axis of the vein was selected. Each bifurcation at the first branch was measured in two orthogonal diameters. The PV bifurcation diameter was defined as the mean of these two measurements, and then the diameter difference was calculated.

### 2.3. Mapping and Ablation Protocol

All the CBA was conducted by experienced operators with more than 100 CBA procedures. All antiarrhythmic drugs (without the use of amiodarone) were discontinued for at least five half‐lives preoperatively. Preprocedural cardiac enhanced computed tomography angiography (CTA) was performed to evaluate the anatomic parameters of PVs and distance between the esophagus and PV antrum. The orientation of PV branches was measured in the transverse plane in the different‐branch CBA group.

The procedure was performed under analgesia obtained with fentanyl. Two thousand IU heparin was administered instantly after the venous access and added to 100 IU/kg body weight after transseptal puncture. Heparinized saline was infused additionally to maintain the activated clotting time at 300–350 s. The transseptal sheath was exchanged over a guidewire for a 15 Fr steerable sheath (Flexcath Advance, Medtronic, Minneapolis, MN). The second‐generation 28 mm CB (Arctic Front Advance, Medtronic) was advanced into the PV with a spiral mapping catheter (Achieve, Medtronic) for support and to map the PV potentials. CBA applications in right PVs were performed under monitoring the diaphragm motion during phrenic nerve pacing in order to avoid phrenic nerve injury.

The CB was advanced in different branches of PVs in 293 patients and in the same branch of PVs in 416 patients as a routine approach. Following the verification of complete occlusion of the PV ostium with a contrast medium injection, a freeze cycle of 120–180 s was applied for one time in each branch. In the different‐branch CBA group, the first applications were performed in the superior branches of superior PVs (SPVs) and in the inferior branches of inferior PVs (IPVs). In the same‐branch CBA group, the applications were performed in the same branch of each PV at least twice. Left common PV (LCPV) with a short trunk (from the ostium to the bifurcation) (< 10 mm) and a big diameter (> 20 mm) was included in this study.

In the majority of the cases, most part of the Achieve catheter was inserted into the bifurcation to obtain stability of the CB catheter and part of the Achieve catheter was at the orifice to record the TTI. In some cases, the Achieve mapping catheter was not positioned proximally enough so as not to sacrifice support and stability of the CB catheter advanced in the branches. The PV potential would be verified after the cryoablation at the same location of the orifice as where the potential was recorded before the cryoablation.

In a cohort of consecutive 30 patients in the different‐branch CBA group, voltage mappings of the LA and PVs with Achieve catheter using a three‐dimensional electroanatomic mapping system (EnSite Velocity system, Abbott) were created in case of sinus rhythm prior to CBA, after the first and second applications in various branches of each PV. In patients with persistent AF, cardioversion was performed prior to the voltage mapping. The total isolated antral surface area was analyzed using a specific software tool of the NavX system.

The end point of ablation was electrical PVI verified by the circular mapping catheter (Achieve, Medtronic, Inc., Minneapolis, MN). The patients who needed additional radiofrequency ablation of PVs after the CBA application were not included in this study.

### 2.4. Follow‐Up

Patients were instructed to continue anticoagulation therapy and antiarrhythmic drugs for at least 3 months. Follow‐up visits were conducted by regular telephone interviews and surface ECGs or 24‐h Holter monitoring when typical symptoms occurred; Class I or III antiarrhythmic drugs were permitted during the blanking period, but not after 90 days. Recurrence was defined according to the patient’s symptoms, and/or if any atrial tachyarrhythmia lasting longer than 30 s was documented beyond a 3‐month blanking period.

### 2.5. Statistical Analysis

Continuous variables were presented as mean ± SD or median with interquartile range, as appropriate. The difference of measurement data between groups was analyzed by independent sample *T*‐test or Mann–Whitney *U*‐test, respectively. Spearman correlation analysis was used to study the correlation between the two variables. Independent variables associated with recurrence of AF were identified by multivariate logistic regression analysis. Kaplan–Meier analysis and log‐rank test were used for survival analysis. A *p* value < 0.05 was considered significant. SPSS software version 19.0 (SPSS, Inc., Chicago, IL) was used for statistical analysis.

## 3. Results

### 3.1. Patient Characteristics

Fifteen patients who had been projected in the different‐branch CBA group while advancement of CB catheter or antral occlusion was not possible in different branches of PVs were not included in this study. In the same‐branch group, 109 patients who were originally projected to be included but actually received PVI in different branches of PVs in parts were not included. When adequate cooling could not be achieved in 56 patients or PV potential could still be recorded in other branch in 53 (10.1%, *p* = 0.163) patients, the Achieve catheter would be changed into the other branch in some PVs. As a result, we enrolled 709 patients with documented nonvalvular AF who underwent initial PVI with a second‐generation CB in our single center, including 293 CBA applications in different branches of PVs and 416 in the same branch of PVs. There were no significant differences in terms of age, gender, type of AF, comorbidities, body mass index, fasting blood glucose, cholesterol, triglyceride, estimated glomerular filtration rate, left and right atrial size, left ventricular size, and left ventricular ejection fraction (*p* > 0.05) between the two groups. Baseline characteristics are shown in Table 1.

**TABLE 1:** Clinical characteristics with respect to different CBA groups.

	Same‐branch CBA group (*n* = 416)	Different‐branch CBA group (*n* = 293)	*Z*/*t*/*χ* ^2^	*p*
Age (years)	61.35 ± 9.55	60.80 ± 9.20	0.778	0.437
Male sex	302 (72.59)	205 (69.97)	0.584	0.445
Persistent atrial fibrillation	151 (36.29)	92 (31.40)	1.831	0.176
Hypertension	305 (73.32)	196 (66.89)	3.421	0.064
Diabetes mellitus	119 (28.61)	74 (18.43)	0.974	0.324
Coronary artery disease	74 (17.79)	39 (13.31)	2.573	0.109
Prior stroke/TIA	110 (26.44)	75 (25.60)	0.064	0.801
Smoking	121 (29.09)	93 (31.74)	0.575	0.448
Drinking	93 (22.36)	69 (23.55)	0.139	0.709
BMI (kg/m^2^)	25.63 ± 3.30	25.33 ± 3.07	1.197	0.232
Fasting blood glucose (mmol/L)	5.57 ± 1.13	5.45 ± 1.05	1.370	0.171
Cholesterol (mmol/L)	4.21 ± 1.06	4.34 ± 1.12	−1.650	0.099
Triglyceride (mmol/L)	1.50 ± 0.98	1.65 ± 1.04	−1.895	0.058
High‐density lipoprotein cholesterol (mmol/L)	1.24 ± 0.25	1.26 ± 0.31	−0.887	0.375
Low‐density lipoprotein cholesterol (mmol/L)	2.34 ± 0.72	2.26 ± 0.72	1.405	0.161
eGFR (mL/min)	93.70 ± 18.55	92.03 ± 18.60	1.177	0.240
LA (mm)	38.54 ± 4.97	39.25 ± 4.95	−1.796	0.073
LV (mm)	48.16 ± 5.43	47.76 ± 4.41	0.936	0.349
Vertical diameter of RA (mm)	39.21 ± 6.66	40.03 ± 5.31	−1.695	0.091
Transverse diameter of RA (mm)	48.05 ± 6.71	48.76 ± 5.95	−1.323	0.186
LVEF (%)	60.35 ± 6.37	61.17 ± 5.56	−1.619	0.106

*Note:* Values are mean ± SD, or *n*/*N* (%); CBA, cryoballoon ablation.

Abbreviations: BMI, body mass index; eGFR, estimated glomerular filtration rate; LA, left atrium; LV, left ventricle; LVEF, left ventricular ejection fraction; PV, pulmonary vein; RA, right atrium; TIA, transient ischemic attacks.

### 3.2. Procedural Results

The fluoroscopy time increased in the different‐branch CBA group. The ablation time and fluoroscopy dose had no significant difference between the two groups. The number of cryoablation of each PV had no significant difference between the two groups. Temperature difference of first and second applications in different branches of each PV had statistical significance in the different‐branch CBA group compared with the control group. During the first application performed in each PV, the recording rate of time to isolation (TTI) and average TTI had no significant difference in each PV separately between the two groups. In the different‐branch CBA group, after the first application in one branch, PV potential could still be recorded in the other branch in 39 patients (13.31%), and the PV foci could trigger AF in 7 of them. Complete PVI was achieved, and sinus rhythm was present in all the cases at the end of the procedure. There was no significant difference in the occurrence of phrenic nerve injury, pericardial effusion, and vagal reflex between the two groups. No other complications were observed in the study. Procedural results are shown in Table 2.

**TABLE 2:** Procedural results with respect to different CBA groups.

	Same‐branch CBA group (*n* = 416)	Different‐branch CBA group (*n* = 293)	*Z*/*t*/*χ* ^2^	*p*
Ablation time (min)	56.2 ± 19.6	58.0 ± 25.9	−1.026	0.305
Fluoroscopy time (min)	22.1 ± 9.5	24.1 ± 8.8	−2.773	0.006
Fluoroscopy dose (mGy)	291.2 ± 209.2	310.7 ± 171.2	−1.291	0.197
Number of LSPV cryoablations	2 (2, 2)	2 (2, 2)	−1.801	0.072
Temperature difference of LSPV branches (°C)	3 (1, 4)	3 (1, 7)	−3.981	< 0.001
Number of LIPV cryoablations	2 (2, 2)	2 (2, 2)	−0.125	0.901
Temperature difference of LIPV branches (°C)	2 (1, 4)	3 (2, 5)	−4.553	< 0.001
Number of RSPV cryoablations	2 (2, 2)	2 (2, 2)	−1.005	0.315
Temperature difference of RSPV branches (°C)	2 (0, 4)	3 (1, 7)	−4.242	< 0.001
Number of RIPV cryoablations	2 (2, 2)	2 (2, 2)	−1.612	0.107
Temperature difference of RIPV branches (°C)	2 (1, 5)	3 (1, 6)	−5.566	< 0.001
Recording of TTI in LSPV	307 (73.8)	218 (74.4)	0.033	0.856
Average TTI in LSPV (S)	42.6 ± 15.4	39.8 ± 11.2	1.121	0.265
Recording of TTI in LIPV	242 (58.2)	175 (59.7)	0.171	0.679
Average TTI in LIPV (S)	40.0 ± 12.8	41.4 ± 13.6	0.586	0.559
Recording of TTI in RSPV	278 (66.8)	196 (66.9)	0.000	0.985
Average TTI in RSPV (S)	46.0 ± 12.3	42.3 ± 18.8	0.212	0.833
Recording of TTI in RIPV	211 (50.7)	169 (57.7)	3.347	0.067
Average TTI in RIPV (S)	40.7 ± 10.5	43.9 ± 11.4	0.286	0.776
Phrenic nerve injury	11 (2.64)	3 (1.02)	2.332	0.127
Pericardial effusion	4 (0.96)	2 (0.68)	0.000	1.000
Vagal reflex	15 (3.61)	6 (2.05)	1.452	0.228

*Note:* Values are mean ± SD, median (25th percentile, 75th percentile), or *n*/*N* (%). CBA, cryoballoon ablation.

Abbreviations: AF, atrial fibrillation; LIPV, left inferior pulmonary vein; LSPV, left superior pulmonary vein; PV, pulmonary vein; RIPV, right inferior pulmonary vein; RSPV, right superior pulmonary vein.

**FIGURE 1:**
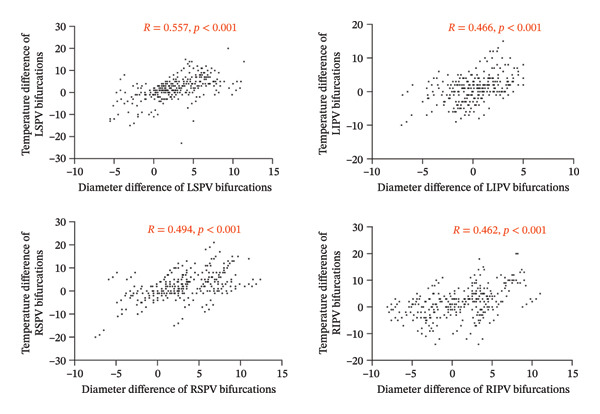
Relationship between the nadir temperature difference and the diameter difference in various branches of each PV. PV, pulmonary vein; LSPV, left superior pulmonary vein; LIPV, left inferior pulmonary vein; RSPV, right superior pulmonary vein; RIPV, right inferior pulmonary vein.

**FIGURE 2:**
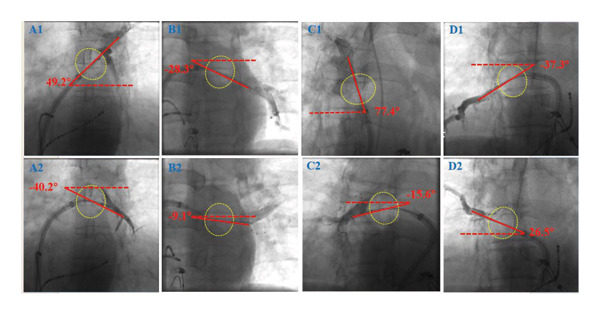
Balloon to PV occlusion was demonstrated by the retention of contrast shown by fluoroscopy in LAO projection (A1, A2, B1, B2) and RAO projection (C1, C2, D1, D2). The CB is highlighted in yellow dotted line. The orientations of CB catheter advanced in the different branches of PVs were measured in the transverse plane. When the CB catheter advanced in the upper branches of LSPV (A1) and RSPV (C1) and lower branches of LIPV (B1) and RIPV (D1), the force directions of the balloon were more biased toward the antrum junction of superior PVs and inferior PVs. When the CB catheter advanced in the lower branches of LSPV (A2) and RSPV (C2) and in the upper branches of LIPV (B2) and RIPV (D2), the roof and bottom antrum would be more liable to be lesioned. LAO, left anterior oblique projection; RAO, right anterior oblique projection; CB, cryoballoon; the other abbreviations as in the legend to Figure 1.

**FIGURE 3:**
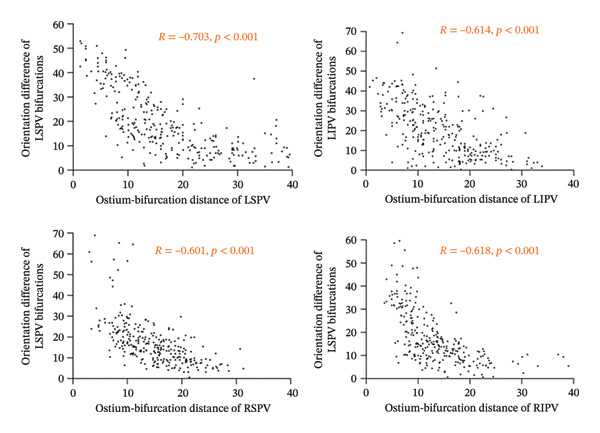
Relationship between the orientation difference in the transverse plane and the ostium–bifurcation distance in various branches of each PV. The abbreviations as in the legend to Figure 1.

**FIGURE 4:**
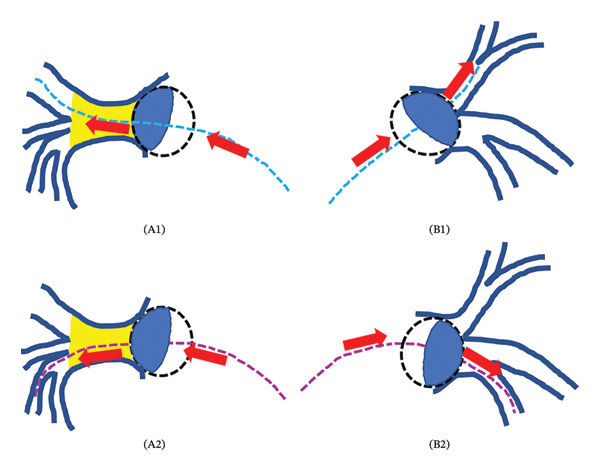
Schematic diagrams of orientation differences of CB catheter in different branches of PV with (A1, A2) and without (B1, B2) long ostium–bifurcation distance. The cryoballoon is highlighted in black, and the PV is demarcated in blue outline. Gray regions represent the long ostium–bifurcation, and blue regions represent freezing surface of the balloon. Blue dotted lines and purple dotted lines represent CB catheter advanced in different branches of PV. The abbreviations as in the legend to Figures 1 and 2.

In the different‐branch CBA group, the cryoballoon’s nadir temperature, diameter, and orientation had significant difference in the upper and lower branches of LSPV and RSPV. The CBA time and orientations were different in the inferior and superior branches of bilateral IPVs. Larger diameters and lower balloon nadir temperatures were detected in the superior branches than in the inferior ones of bilateral SPVs, while the differences of diameter and balloon nadir temperature were not significant in various branches of IPVs (Table 3). The nadir temperatures were all moderately correlated with the branch diameters of the four PVs (*p* < 0.001). The temperature differences were positively correlated with the diameter differences in various branches of each PV (Figure 1). The orientations of CB catheter advanced in the upper and lower branches of each PV were significantly different in the transverse plane (Table 3, Figure 2). The orientation difference in the transverse plane between the upper and lower branches was correlated negatively with the ostium–bifurcation distance (Figures 3 and 4). Nineteen patients (6.5%) had LCPV with a short trunk and a large diameter; CBA was performed in different branches of each PV branch separately.

**TABLE 3:** Procedural data of cryoablation in different bifurcations of each PV.

	**LSPV S**	**LSPV I**	***Z***	**p**

Balloon nadir temperature (−°C)	50 (45∼54)	48 (44∼51)	−4.844	< 0.001
Ablation time (S)	180 (120∼180)	150 (130∼180)	−0.993	0.321
Diameter of branch (mm)	10.5 (8.7∼13.1)	6.2 (5.3∼7.7)	−10.627	< 0.001
Orientation of branch (°)	34.6 (23.9∼42.1)	15.8 (9.6∼30.6)	−11.204	< 0.001

	**LIPV S**	**LIPV I**		

Balloon nadir temperature (−°C)	43 (39∼46)	43 (40∼45)	−1.727	0.084
Ablation time (S)	180 (180∼180)	180 (180∼180)	−4.484	< 0.001
Diameter of branch (mm)	8.6 (7.1∼10.2)	8.9 (7.6∼10.5)	−1.613	0.107
Orientation of branch (°)	18.3 (3.3∼20.4)	−5.4 (−16.0∼5.6)	−14.191	< 0.001

	**RSPV S**	**RSPV I**		

Balloon nadir temperature (−°C)	52 (47∼54)	51 (46∼53)	−2.714	0.007
Ablation time (S)	180 (120∼180)	150 (120 ∼180)	−1.698	0.089
Diameter of branch (mm)	12.0 (9.8∼14.7)	7.5 (5.8∼9.4)	−7.160	< 0.001
Orientation of branch (°)	47.8 (36.9∼55.4)	37.3 (25.0∼50.7)	−18.194	< 0.001

	**RIPV S**	**RIPV I**		

Balloon nadir temperature (−°C)	45 (41∼52)	47 (40∼49)	−1.844	0.065
Ablation time (S)	180 (180∼180)	180 (150∼180)	−3.561	< 0.001
Diameter of branch (mm)	8.0 (5.6∼9.8)	8.1 (6.4∼10.7)	−0.855	0.398
Orientation of branch (°)	11.1 (1.8∼20.4)	−2.6 (−19.2∼8.9)	−13.214	< 0.001

*Note:* S, superior branch; I, inferior branch.

Abbreviations: LIPV, left inferior pulmonary vein; LSPV, left superior pulmonary vein; PV, pulmonary vein; RIPV, right inferior pulmonary vein; RSPV, right superior pulmonary vein.

**FIGURE 5:**
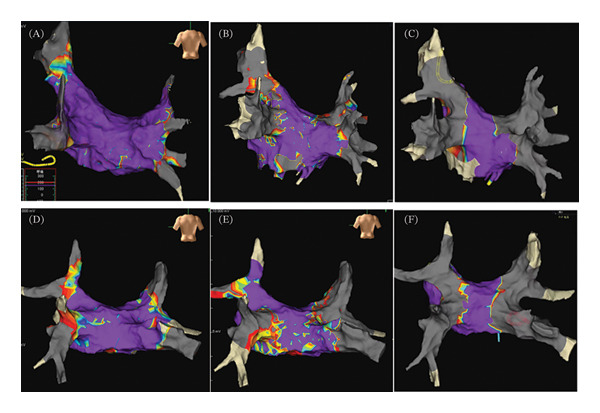
Posterior view of LA voltage mapping created using the EnSite NavX system in two patients ((A–C) and (D–F), respectively). Low‐voltage areas are defined as < 0.2 mV, purple regions represent normal myocardium. (A, D): Voltage mappings prior to CBA; (B, E): voltage mappings after the first application in one branch of each PV; (C, F): voltage mappings after the second application in the other branch of each PV. The total isolated antral surface area after the application in a second branch was larger than that after the first application. LA, left atrial; CBA, cryoballoon ablation; PV, pulmonary vein.

**FIGURE 6:**
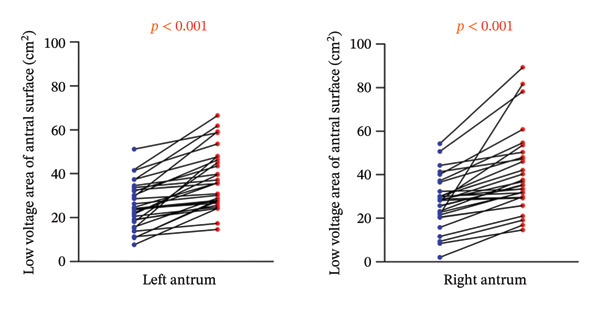
Comparison of low‐voltage area of antral surface in the LA after the first (blue dot) and second applications (red dot) in various branches of LPV and RPV. LA, left atrium; PV, pulmonary vein; LPV, left pulmonary vein and the antrum; RPV, right pulmonary vein and the antrum.

In a consecutive 30 patients, voltage mappings of the LA and PV were analyzed in the case of sinus rhythm prior to CBA, after the first and second applications in various branches of each PV. The total isolated antral surface areas after the ablation in a second branch were larger than those after the first ablation whether in left PVs ([37.07 ± 13.48] cm^2^ vs [25.30 ± 10.58] cm^2^, *p* < 0.001) or in right PVs ([40.40 ± 18.37] cm^2^ vs [27.60 ± 11.84]  cm^2^, *p* < 0.001) (Figures 5 and 6).

### 3.3. Follow‐Up

**FIGURE 7:**
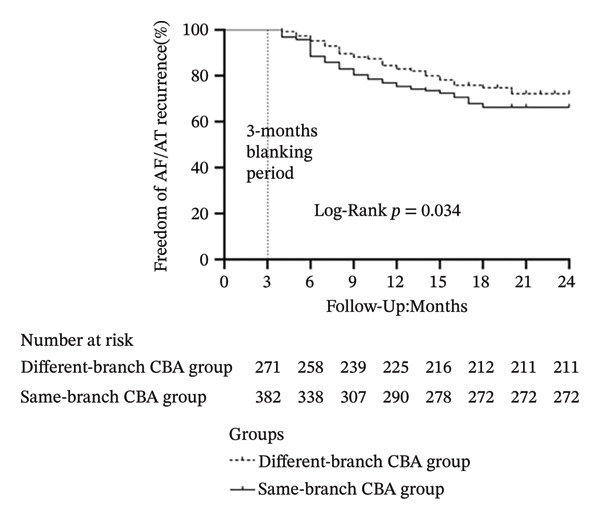
Freedom of atrial arrhythmia recurrence in the different‐branch CBA group and the same‐branch CBA group at a 2‐year follow‐up. Kaplan–Meier analysis and log‐rank test were used for survival analysis.

A total of 271 (92.49%) patients in the different‐branch CBA group and 382 (91.83%) patients in the same‐branch CBA group were followed by regular telephone interviews and surface ECGs or 24‐h Holter monitoring when typical symptoms occurred. Regardless of the 90‐day blanking period, the recurrence rate of atrial arrhythmia was statistically lower in the different‐branch CBA group compared with that in the same‐branch CBA group (*p* = 0.034) at a 2‐year follow‐up (Figure 7).

Multivariate logistic regression analysis was conducted to identify independent variables associated with recurrence of AF in the different‐branch CBA group (Table 4). Orientation difference of LSPV branches (OR = 0.912; *p* < 0.001), BMI (OR = 0.853; *p* < 0.001), and early recurrence within the 3‐month blanking period (OR = 17.636; *p* < 0.001) were statistically significant predictors of AF recurrence.

**TABLE 4:** Multivariate analysis of independent variables associated with recurrence of AF.

	*β*	Wald *χ* ^2^	*p*	Odds ratio	95% confidence interval	
					Lower limits	Upper limits
Gender (Male)	0.608	2.398	0.121	1.838	0.851	3.969
Age	−0.023	0.835	0.361	0.977	0.929	1.027
Persistent atrial fibrillation	0.28	0.303	0.582	1.324	0.488	3.591
Orientation difference of LSPV bifurcations	−0.092	22.345	< 0.001	0.912	0.878	0.947
Orientation difference of LIPV bifurcations	0.024	3.251	0.071	1.025	0.998	1.052
Orientation difference of RSPV bifurcations	−0.001	0.003	0.958	0.999	0.971	1.028
Orientation difference of RIPV bifurcations	0.021	1.424	0.233	1.021	0.987	1.057
LCPV	−0.258	0.159	0.69	0.773	0.218	2.742
BMI	−0.159	12.956	< 0.001	0.853	0.783	0.930
Left atrial diameter	0.056	2.606	0.106	1.057	0.988	1.131
Early recurrence	2.87	37.303	< 0.001	17.636	7.021	44.297

Abbreviations: AF, atrial fibrillation; BMI, body mass index; LCPV, left common pulmonary vein; LIPV, left inferior pulmonary vein; LSPV, left superior pulmonary vein; RIPV, right inferior pulmonary vein; RSPV, right superior pulmonary vein.

## 4. Discussion

To our knowledge, this is the first study to advance the CB in different branches of PVs as a routine approach during AF cryoablation. The fluoroscopy time increased in the different‐branch CBA group. The ablation time and fluoroscopy dose had no significant difference between the two groups. Larger diameters and lower balloon nadir temperatures were detected in the superior branches than in the inferior ones of bilateral SPVs. The nadir temperatures were correlated moderately with the branch diameters of the four PVs, and the nadir temperature differences were correlated positively with the diameter differences in various branches of each PV. Large PVs would allow the CB to advance distally, while the PV antrum and the PV junction near LA anterior/posterior wall remained intact. When we advanced the CB in a different branch of PV, which had a smaller diameter, the proximal part of the balloon positioned closer to the central LA and more blood rewarming flow surrounding the inflated balloon. The CBA would not be terminated prematurely due to a deep position of balloon and a rapid temperature reduction below −55°C, and thus a wide area of antral ablation lesion might be created.

In the present study, the orientation differences of CB catheter advanced in different branches of each PV were correlated negatively with the ostium–bifurcation distances. In general, the first application was performed in the superior branch of SPV, and in the inferior branch of IPV. In PVs with early bifurcation, when the CB catheter advanced in the upper branches of bilateral SPVs and lower branches of bilateral IPVs, the force directions of the balloon were more biased toward the antrum junctions of SPVs and IPVs. When the CB catheter advanced in the lower branches of bilateral SPVs and in the upper branches of bilateral IPVs, the roof and bottom antrum would be more liable to be lesioned.

As shown in Figure 4, the orientation of CB catheter in different branches of PV with a long ostium–bifurcation distance was mainly determined by the orientation of main trunk. As a result, when the catheter was inserted into different branches at the distal end, the angle difference was small. On the contrary, in those with a short ostium–bifurcation distance, the orientation of CB catheter in different branches depended directly on the orientation of each branch and the orientation difference would be more obvious. Since the orientation differences between different branches were negatively correlated with the ostium–bifurcation distances (Figure 3), CB catheter advanced in different branches of PVs with long ostium–bifurcation distances had less significance to achieve different force directions of CB.

An LCPV has been defined as having a distance of 5 mm or more from the left PV ostium to the bifurcation [8]. Kubala et al. reported that there were more AF‐free patients with a normal PV anatomy compared to those with an LCPV after the PVI with the CBA [9]. They speculated that the difficulties in catheter manipulation resulted in insufficient circumferential lesion formation. In the different‐branch CBA group, 19 patients (6.5%) who had an LCPV with a short trunk and a large diameter were included and CBA was performed in different branches of each PV to achieve a sufficient circumferential lesion. Furthermore, LCPV was not confirmed to be a predictor of AF recurrence in the multivariate logistic regression analysis.

TTI, which was observed by loss of PV potentials and conduction block, when available, is the best indicator of isolated lesion creation, and it is proven that earlier TTI is related to durable PVI [10–12]. In this study, the record rate of TTI was not very high, for in some cases, the Achieve mapping catheter was not positioned proximally enough so as not to sacrifice the support and stability of the CB catheter advanced in the branches. Long‐term PVI was demonstrated if TTI was less than 60 s [10, 12, 13]. Therefore, when TTI was achieved in 60 s, single‐freeze application was recommended [7]. When TTI was not visualized or more than 60 s, a bonus freeze may be appropriate [14]. In our study, the CB was advanced in different branches of each PV and a freeze cycle of 120–180 s was applied for one time in each branch. The reduced freeze duration was mainly determined by a rapid temperature reduction below −55°C or a close distance between the esophagus and PV antrum with a temperature below −50°C, whatever the TTI was. Moreover, a short duration of TTI might also indicate a deep position of the balloon in PVs. After the first application in one branch of each PV, although PVI had been achieved in this branch, PV potentials could still be recorded in the other branch in 39 patients, and it was confirmed to be the PV foci that could trigger AF in 7 of them. From this point of view, CB catheter advanced in different branches would lead to eliminate PV foci adequately; additionally, after PVI of one branch of individual PV, detection of the other branch might be considered.

Several trials revealed that CB‐based PVI was noninferior to RF‐based PVI for patients with respect to the primary efficacy and safety end points [2, 15, 16]. It was presumably because of the high durability of the PVI after the CB ablation [17, 18]. A meta‐analysis and systematic review found that RFCA and CBA offered similar durability of PVI lesions [19]. Miyazaki et al. reported that the area encircled by circumferential PVI using the RF catheter was significantly larger than the PVI area after ablation using the second‐generation CB during the chronic phase [20]. A wide ablation area will eliminate more arrhythmic foci and contribute to better outcomes. A segmental CBA procedure will ensure a wide circumferential lesion area, while it is difficult to confirm a continuous lesion without conventional three‐dimensional electro‐anatomical mapping. Multiple studies have clarified that PV antrum isolation has advantage over segmental PVI with respect to the AF freedom post‐procedure [5, 6, 21]. Empirical extra PV ablation strategies for substrate modification have been applied to AF patients [22, 23]. However, extra‐PV CB applications at the antrum raise concern of collateral damage near the posterior LA [24, 25]. CBA of LA roof in persistent AF did not systematically explore the isolation area or potential complications [26]. In our experience, most of the CB applications in different branches of each PV could obtain a complete occlusion of the PV ostium. In the latest consecutive 30 patients who underwent voltage mapping, the total isolated antral surface areas after the applications in a second branch were larger than those after the first applications. Furthermore, multivariate logistic regression analysis revealed that a small orientation difference of LSPV branches was a predictor of AF recurrence. A sufficient ablation region might be created in the patients with big orientation difference of PV branches.

### 4.1. Limitations

This study has some limitations. First, this study was conducted by experienced operators, and the significance of this technique needs to be further evaluated in view of the variability in patient selection criteria, operator technique, and PV anatomy. Second, the three‐dimensional mapping system was not used for all the patients due to time‐consuming process and economic reason. Third, the fluoroscopy time increased in the different‐branch CBA group. The ablation time and fluoroscopy dose had no significant difference between the two groups. This may be due to the variability in operator experience, and a parallel controlled study will be necessary.

## 5. Conclusions

CBA applications performed in different branches of each PV could create a wide isolated antral surface area and eliminate PV foci adequately. CBA in different branches of PVs with long ostium–bifurcation distances had less significance. The recurrence rate of atrial arrhythmia was statistically lower in the different‐branch CBA group compared with that in the same‐branch CBA group.

## Funding

This study was supported by the Key Research and Development Project of the Joint Plan of Liaoning Province Science and Technology Plan, 2025JH2/101800030.

## Disclosure

The funding source had no role in the design of the study, collection, analysis, or interpretation of data, or in the writing of the manuscript or the decision to submit it for publication.

This study was previously presented as a poster at the 33rd Great Wall International Congress of Cardiology & Asian Heart Society Congress 2022, with the poster information available at ScienceOpen [27].

## Conflicts of Interest

The authors declare no conflicts of interest.

## Data Availability

The data that support the findings of this study are available upon request from the corresponding author. The data are not publicly available due to privacy or ethical restrictions.
